# Echocardiographic predictors of symptomatic cardiotoxicity among patients undergoing chemotherapy: A systematic review and meta-analysis

**DOI:** 10.1097/MD.0000000000029562

**Published:** 2022-07-15

**Authors:** Muhammad Umer Siddiqui, Youssef Yaacoub, Heidi-Anne Hanson, Joey Junarta, Ahmed K. Pasha, Mahek Shah

**Affiliations:** a Internal Medicine, Thomas Jefferson University Hospital, Philadelphia, PA; b Internal Medicine, Catholic Medical Center, Manchester, NH; c Internal Medicine, Lake Region General Hospital, Laconia, NH; d Vascular Medicine, Mayo Clinic Health System, Rochester, MN; e Cardiovascular Medicine, Thomas Jefferson University Hospital, Philadelphia, PA.

**Keywords:** cardiotoxicity, chemotherapy, echocardiogram, ejection fraction, global longitudinal strain

## Abstract

**Background:**

Chemotherapeutic agents have been associated with cardiotoxicity; thus, they require close monitoring. Several echocardiographic variables have been investigated as early predictors of symptomatic cardiotoxicity in patients undergoing chemotherapy.

**Objective:**

To identify if global longitudinal strain (GLS) is a better predictor of symptomatic cardiotoxicity compared to left ventricular ejection fraction (LVEF) in patients receiving chemotherapy.

**Methods:**

MEDLINE, Scopus, and Cochrane Central Register of Controlled Trials were searched from inception through December 2020. Adults who developed symptomatic cardiotoxicity (New York Heart Association [NYHA] Class III–IV heart failure, cardiac arrest, or cardiac death) after undergoing chemotherapy with pre- and postchemotherapy echocardiographic measures of cardiac function were included. The primary focus was on the prediction of symptomatic cardiotoxicity. Estimates were reported as random effects hazard ratios (HR) with 95% confidence intervals (CI).

**Results:**

Four studies met inclusion criteria. The most common malignancy identified in the included studies was breast cancer, and the most common chemotherapeutic agent utilized was anthracyclines. Most studies utilized the Simpson biplane method to measure echocardiographic parameters. Pooled results demonstrated no significant association between LVEF and the prediction of symptomatic cardiotoxicity (HR 1.48; 95% CI, 0.96–2.27; *P* = 0.07). However, 2 studies that analyzed GLS found it to be a strong predictor of symptomatic cardiotoxicity (HR 1.46; 95% CI, 1.34–1.58; *P* < .001). There was no significant association between symptomatic cardiotoxicity and baseline left ventricular end diastolic volume, end systolic volume, or end diastolic volume index.

**Conclusions:**

GLS may predict symptomatic cardiotoxicity and be used to monitor patients on chemotherapy for symptomatic cardiac dysfunction. While the pooled results for baseline LVEF identified that it is not a predictor of symptomatic cardiotoxicity, this differs from the findings of the only randomized trial included in this meta-analysis. The data for baseline GLS as a predictor of symptomatic cardiotoxicity is encouraging, but definite evidence that GLS may be superior to LVEF is lacking. Prospective randomized, blinded trials are required to identify if 1 echocardiographic parameter may be superior to the other.

## 1. Introduction

In 2016, there were more than 10 million cancer survivors in the United States, which is expected to double by 2026 approximately.^[1]^ Certain chemotherapeutic agents are associated with cardiotoxicity, which leads to their limited use in eligible candidates. Left ventricular ejection fraction (LVEF) is routinely used to monitor cardiac function in patients undergoing chemotherapy.^[2]^ However, various studies have shown that by the time the LVEF is reduced, patients may have already suffered from irreversible damage. Cardinale et al found that among patients treated with anthracyclines and diagnosed with impairment in LVEF, 36% of patients failed to recover to normal LV function, often despite therapeutic interventions.^[3]^ Similarly, Tan et al reported that among patients treated with anthracyclines, taxanes, and trastuzumab, who developed LV dysfunction, the abnormality generally persisted for greater than 2 years.^[4]^

There is a lack of consensus on how to define cardiotoxicity. The American Society of Echocardiography defines cardiotoxicity as >10% decline in LVEF to a final value of <53% or >15% relative decline in global longitudinal strain (GLS).^[5]^ Whereas the National Cancer Institute includes symptoms of LV dysfunction in their definition and divides the dysfunction into 5 grades, with grade 2 suggesting mild heart failure symptoms and grade 5 involving death.^[6]^ Once symptoms persist due to anthracycline-induced heart failure, the prognosis is poor, with <50% survival at 1 year.^[7]^ Thus, it has been proposed that merely relying on LVEF may not identify the insidious remodeling that results in symptomatic cardiotoxicity.^[8]^ Studies have also suggested that myocardial deformation and GLS might be more sensitive than LVEF and may be able to detect chemotherapy-induced symptomatic cardiotoxicity earlier.^[9,10]^ Early reductions in peak longitudinal strain (PLS) and strain rate while the LVEF remains normal may predict further decreases in systolic function of the left ventricle.^[11,12]^

A recent meta-analysis by Oikonomou et al identified GLS as having a good prognostic performance for subsequent cancer therapy-related cardiac dysfunction.^[13]^ However, Oikonomou et al incorporated studies that included patients with both symptomatic and asymptomatic cardiotoxicity. It remains unclear whether a drop in GLS among patients receiving chemotherapy will predict eventual symptomatic cardiotoxicity. We, therefore, conducted a systematic review and meta-analysis to identify echocardiographic predictors of symptomatic cardiotoxicity among chemotherapy patients to fill this knowledge gap.

## 2. Methods

### 2.1. Data sources and search strategy

According to the Preferred Reporting Items for Systematic Review and Meta-Analyses (PRISMA) guidelines, this systematic review and meta-analysis was reported.^[14]^ Medline, Scopus, and Cochrane Central Register of Controlled Trials were searched from database inception through December 2020 using the following combination of keywords: Echocardiography OR global longitudinal strain OR left ventricular function OR myocardial strain OR echocardiographic characteristics AND chemotherapy OR anthracyclines AND left ventricular dysfunction OR myocardial ischemia OR myocardial infarction OR cardiac events OR cardiac death OR heart failure. No time restriction was placed on the search; however, the language was restricted to English. Online libraries including www.clinicaltrialresults.org, www.clinicaltrials.gov, abstracts, and presentations from major cardiovascular proceedings were also searched to identify gray literature. The Supplementary Material (Supplemental Figure 1, http://links.lww.com/MD/G899) outlines a complete description of the search strategy. All citations retrieved from the search were transferred to EndNote X7.5 (Thompson ISI ResearchSoft, Philadelphia, PA) Reference Manager, and duplicates were removed. As this is a meta-analysis, this study did not require approval by our institutional review board.

### 2.2. Study selection

All citations were screened by 2 independent reviewers (MUS and YY) based upon eligibility criteria. Inclusion criteria include identifying adults who developed symptomatic cardiotoxicity after undergoing chemotherapy with pre-and postchemotherapy echocardiographic measures of cardiac function. Symptomatic cardiotoxicity was defined by the presence of signs and New York Heart Association (NYHA) Class III-IV symptoms of congestive heart failure (shortness of breath, swelling, paroxysmal nocturnal dyspnea), cardiac arrest, or cardiac death. No restrictions to the type of malignancy were placed. Studies that did not report the relation of echocardiographic measures to cardiotoxicity were excluded. nonrelevant citations were excluded based on title and abstract. Abstracts, case reports, and editorials were also excluded. Both randomized controlled trials (RCTs) and observational studies (OS) were included. Any disagreements between the 2 investigators were resolved by consensus of the study group.

### 2.3. Data extraction and risk of bias

Two independent reviewers (MUS and YY) extracted the data regarding the year of publication, sample size, study design, baseline patient profiles, echocardiographic parameters, type of malignancy, type of chemotherapy agent, and follow-up time using a standardized data extraction form. Echocardiographic parameters included were GLS, LVEF, left ventricular end-diastolic volume (LVEDV), left ventricular end-diastolic volume index (LVEDVI), left ventricular end-systolic volume (LVESV), left ventricular end-systolic volume index (LVESVI), and average segmental longitudinal strain. The risk of bias was assessed using the Cochrane risk of bias tools for RCTs and SIGN methodology for observational studies. Each potential source of bias was graded as high, low, or unclear.

### 2.4. Statistical analysis

Outcomes from each study were pooled and compared using a random-effects model to account for potential between-study variance. The treatment effect was reported as hazard ratios (HR) with 95% confidence intervals (CI). The I^2^-statistic was quantified to measure heterogeneity, with values >25%, 50%, and 75% consistent with low, moderate, and high degrees of heterogeneity, respectively.^[15]^ Review Manager Software v5.3 was used for all analyses. Publication bias was not assessed since the number of studies was low (<10). *P* values <0.05 were considered statistically significant. Certainty in the evidence (i.e., confidence in the final estimates) was assessed using the GRADE approach (Grades of Recommendation, Assessment, Development, and Evaluation) based on the risk of bias, imprecision, indirectness, inconsistency, and publication bias.^[16]^

## 3. Results

### 3.1. Baseline demographics

**Figure 1.**
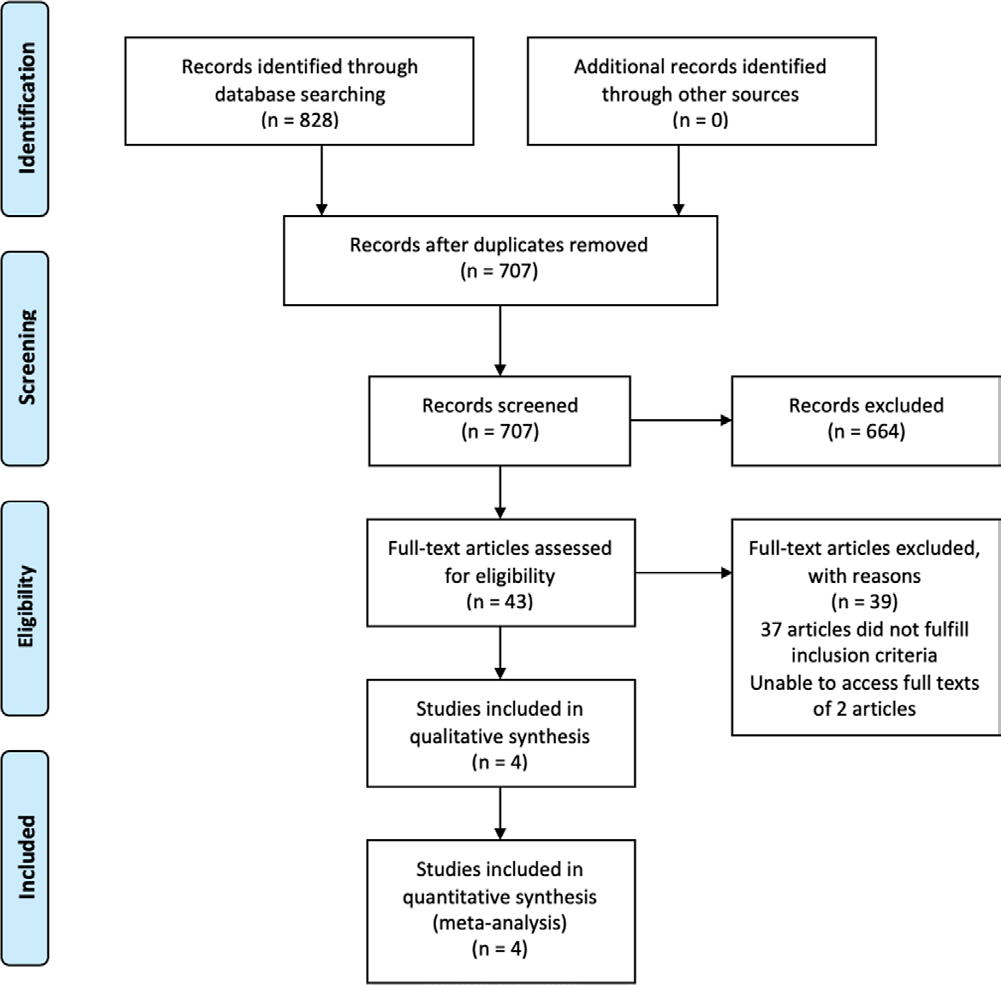
The Preferred Reporting Items for Systematic Reviews and Meta-Analyses (PRISMA) flow diagram of the included studies.

**Figure 2.**
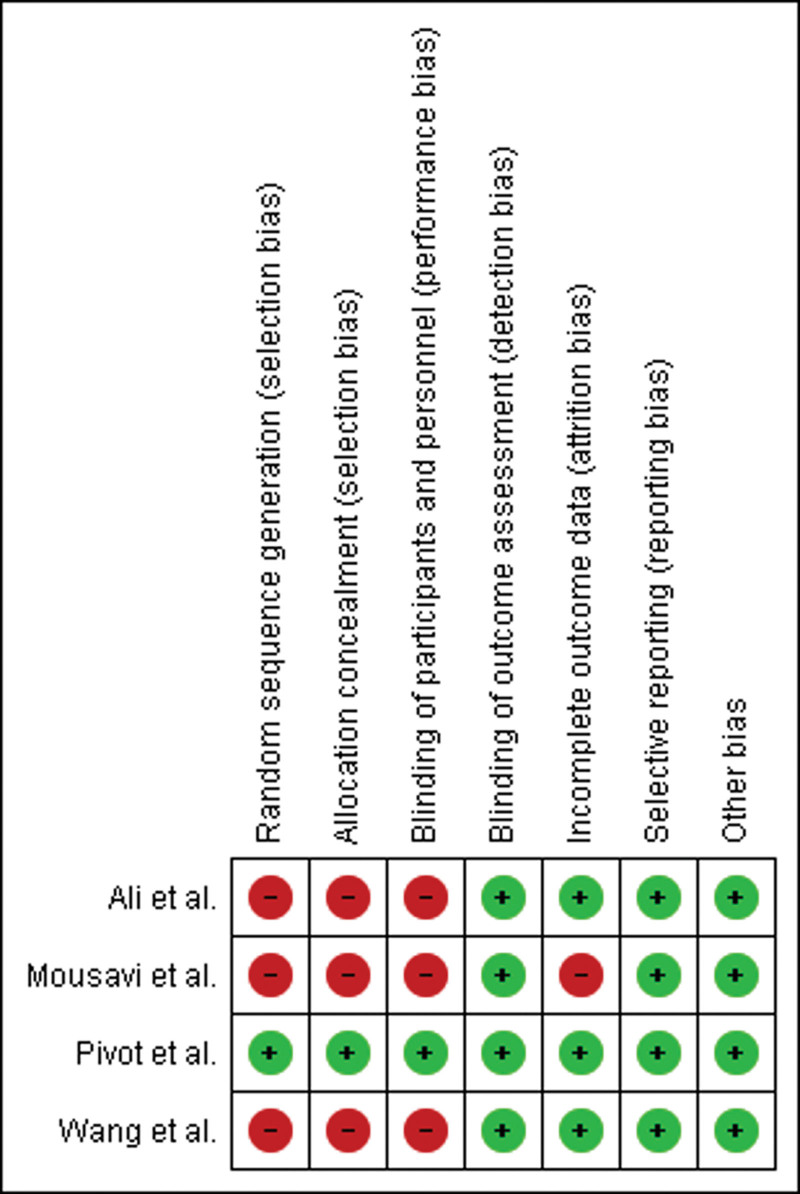
Risk of bias summary for the included studies.

Of 708 unique articles screened, 4 studies were included in the analysis. The systematic review and study selection are outlined in the PRISMA flow chart (Fig. 1). Among the 4 included studies, only 1 was an RCT. A summary of the included studies is shown in Table 1. The mean follow-up period was 7.3 years. The most common echocardiographic parameter measured was LVEF. The baseline LVEF in patients with and without symptomatic cardiotoxicity was >50%. The baseline GLS for patients with symptomatic cardiotoxicity varied from –15 to –16, whereas the baseline GLS in patients without symptomatic cardiotoxicity varied from –17.8 to –19.7. Supplemental Table 1, http://links.lww.com/MD/G899 and Figure 2 show the risk of bias assessment, and all studies had a low risk of bias overall. However, the risk of selection bias was high among OS due to a lack of randomization. Similarly, the risk of performance bias was high among OS due to a lack of blinding.

**Table 1:** Summary of included studies.

Author, year	Study design	Inclusion criteria/baseline LVEF & GLS	Primary endpoint	Echocardiogram technique/variables	Type of cancer	Chemotherapy regimen	Follow-up duration
Ali, 2016	Retrospective observational study	Patients with hematologic cancers treated with anthracycline who underwent prechemotherapy echocardiography/ Baseline LVEF: 62 (No MACE), 58 (MACE) & GLS: -19.7 (No MACE), -15 (MACE)	Symptomatic heart failure or cardiac death	Simpson biplane method/LVEFLVEDVLVEDVILVESVLVESVIGLSASLS	Leukemia or lymphoma	Anthracyclines	4.2 years
Mousavi, 2015	Prospective blinded observational study	Patients treated with anthracyclines with a baseline LVEF between 50 and 59% by 2D echocardiography/Baseline LVEF: 54 (No MACE), 53 (MACE) & GLS: -17.8 (No MACE), -16 (MACE)	NYHA class III or IV CHF, cardiac arrest, or cardiac death	Modified Biplane Method and 2D CPA/LVEFLVEDVLVEDVILVESVLVESVIGLS	Multiple cancers	Anthracyclines	10 years
Pivot, 2015	Open-label phase 3 randomized noninferiority trial	12- to 6-months of trastuzumab given either concomitantly or sequentially after standard (neo)-adjuvant chemotherapy in women with HER2-positive early breast cancer/ Baseline LVEF: >50%	Cardiac death and NYHA class III or IV CHF	Not Reported/LVEF	Breast cancer	Anthracycline + Trastuzumab	5 years
Wang, 2015	Retrospective observational study	Patients with cancer and underwent chemotherapy/ Baseline LVEF: 64 (MACE), 68 (No MACE)	New York Heart Association class III or IV CHF, cardiac arrest, or cardiac death	Visual/LVEFLVIDLVIS	Multiple cancers	Anthracyclines	10 years

ASLS = average segmental longitudinal strain, CHF = congestive heart failure, GLS = global longitudinal strain, LVEDV = left ventricular end diastolic volume, LVEDVI = left ventricular end diastolic volume index, LVEF = left ventricular ejection fraction, LVESV = left ventricular end systolic volume, LVESVI = left ventricular end systolic volume index, MACE = major adverse cardiovascular events, NYHA = New York Heart Association, PSLS = peak systolic longitudinal strain.

## 4. Predictors of symptomatic cardiotoxicity

### 4.1. Baseline left ventricular ejection fraction

All 4 trials analyzed LVEF as a predictor of symptomatic cardiotoxicity. The Simpson biplane method was the most utilized technique to measure LVEF. Two studies reported that LVEF could be used to predict cardiotoxicity in patients receiving chemotherapy.^[17,18]^ Two studies did not show any significant relationship between LVEF and prediction of symptomatic cardiotoxicity (Supplemental Table 2, http://links.lww.com/MD/G899).^[9,19]^

**Figure 3.**
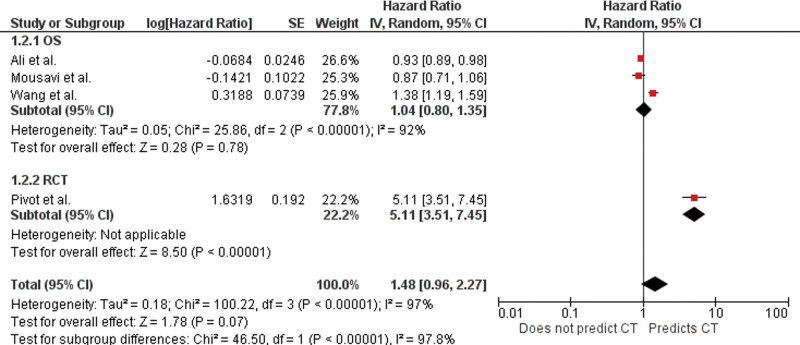
Forest plot for left ventricular ejection fraction as a predictor of symptomatic cardiotoxicity with pooled hazard ratios and study types. The pooled hazard ratio (HR) with 95% confidence interval (CI) were calculated using a random effects model. Weight refers to the contribution of each study to the pooled estimate. Squares and horizontal lines denotes the point estimate and 95% CI for each study’s HR. The diamond signifies the pooled HR; the diamond center denotes the point estimate and the width denotes the 95% CI. CT = cardiotoxicity, OS = observational study, RCT = randomized controlled trial.

The pooled analysis did not demonstrate any significant association between LVEF and prediction of symptomatic cardiotoxicity (HR 1.48; 95% CI, 0.96–2.27; *P* = .07). The I^2^ value was 97%, indicating an increased level of heterogeneity among the studies (Fig. 3).

The sensitivity analysis of the pooled findings after the exclusion of the RCT data conducted by Pivot et al showed results consistent with the overall HR of the baseline LVEF (1.80, 95% CI 0.80–1.35) (Fig. 3).^[18]^ Interestingly, when evaluated individually, the trial conducted by Pivot et al was significant for baseline LVEF as a predictor of symptomatic cardiotoxicity. This is likely due to a lower baseline LVEF (50–59%) of the participants in the study and the addition of trastuzumab to anthracyclines for the treatment of breast cancer, which in itself is cardiotoxic.^[20]^

### 4.2. Baseline global longitudinal strain

**Figure 4.**
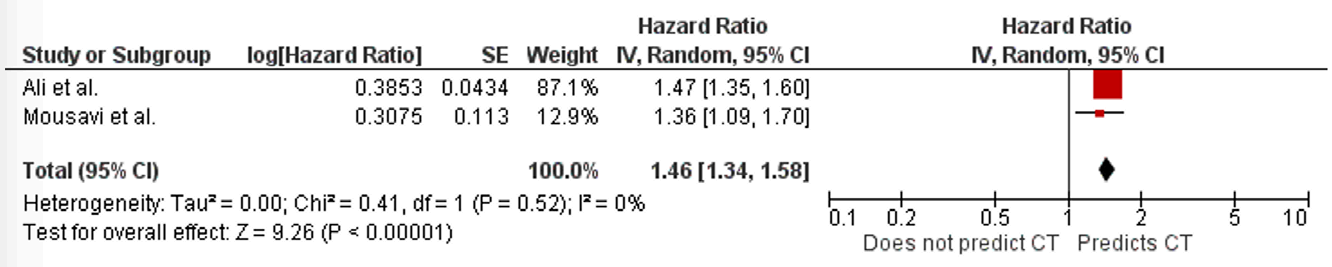
Forest plot for global longitudinal strain as a predictor of symptomatic cardiotoxicity with pooled hazard ratios. CT = cardiotoxicity.

Two studies reported baseline GLS prior to initiating chemotherapy.^[9,19]^ Both studies found GLS to be a significant predictor of symptomatic cardiotoxicity in patients receiving chemotherapy. Data for meta-analysis was pooled from the 2 studies. The meta-analysis demonstrated a significant association between GLS and prediction of symptomatic cardiotoxicity (HR 1.46; 95% CI, 1.34–1.58; *P* ≤ 0.001) (Fig. 4). The I^2^ value was noted to be 0, indicating no heterogeneity.

### 4.2. Baseline left ventricular end diastolic volume

**Figure 5.**
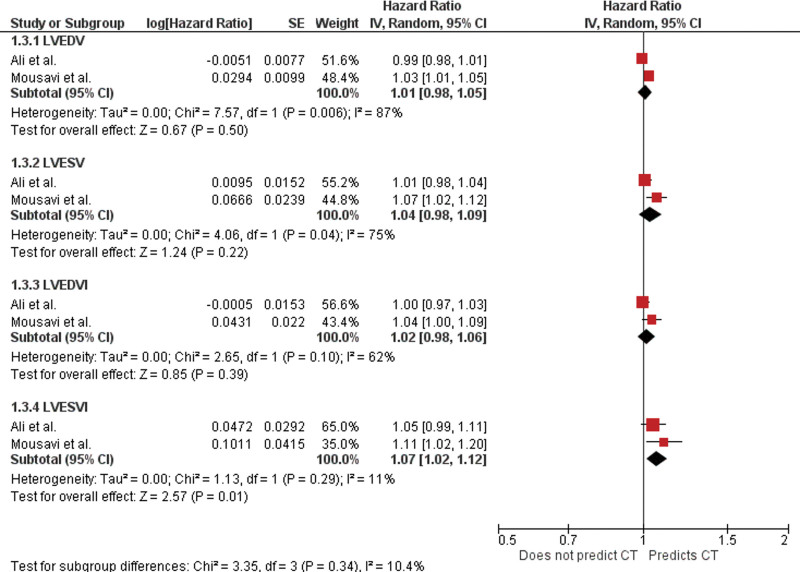
Forest plot for LVEDV, LVESV, LVEDI and LVESVI as a predictor of symptomatic cardiotoxicity showing individual and pooled hazard ratios. CT = cardiotoxicity, LVEDI = left ventricular end diastolic volume index, LVEDV = left ventricular end diastolic volume, LVESV = left ventricular end systolic volume, LVESVI = left ventricular end systolic volume index, .

Two studies reported baseline LVEDV.^[9,19]^ Ali et al did not find any association between LVEDV and subsequent cardiac events (HR 0.99; 95% CI, 0.98–1.01; *P* = .580). In contrast, Mousavi et al found a significant association between LVEDV and symptomatic cardiotoxicity (HR 1.03; 95% CI, 1.01–1.05; *P* = .012). The pooled analysis did not identify any significant association (Fig. 5).

### 4.3. Baseline left ventricular end systolic volume

Two studies reported baseline LVESV, and the results were contrasting.^[9,19]^ Ali et al found LVESV not to be a predictor of cardiotoxicity (HR 1.01; 95% CI, 0.98–1.04; *P* = .370), whereas Mousavi et al found LVESV to be associated with the development of symptomatic cardiotoxicity (HR 1.07; 95% CI, 1.02–1.12; *P* = .0029). The pooled analysis did not identify any significant association (Fig. 5).

### 4.3. Baseline left ventricular end diastolic volume index

Two studies reported LVEDVI.^[9,19]^ Ali et al did not find any association between LVEDVI and occurrence of symptomatic cardiotoxicity (HR 1.00; 95% CI, 0.97–1.03; *P* = .910). In contrast, Mousavi et al found LVEDVI to be associated with the occurrence of symptomatic cardiotoxicity (HR 1.04; 95% CI, 1.00–1.09; *P* = .039). The pooled analysis did not identify any significant association (Fig. 5).

### 4.4. Baseline left ventricular end systolic volume index

Two studies reported this echocardiographic parameter.^[9,19]^ Ali et al did not find a significant association between LVESVI and the occurrence of symptomatic cardiotoxicity (HR 1.05; 95% CI, 0.99–1.11, *P* = .058), whereas Mousavi et al found LVESVI to be associated with the occurrence of symptomatic cardiotoxicity (HR 1.11; 95% CI, 1.02–1.20, *P* = .012). The pooled analysis did identify a significant association (Fig. 5).

### 4.5. Other 2D transthoracic echocardiographic parameters

Other echocardiographic parameters examined in association with symptomatic cardiotoxicity included average segmental longitudinal strain (ASLS), left ventricular internal diameter end diastole (LVID), and left ventricular internal diameter end-systole (LVIS). Ali et al observed that ASLS had a significant association with cardiotoxicity development (HR 1.85; 95% CI, 1.65–2.08, *P* < .001.^[9]^ Wang et al found LVID and LVIS to be associated with symptomatic cardiotoxicity (LVID: HR 1.38; 95% CI, 1.08–1.76; *P* ≤ 0.0001; LVIS: HR 1.50; 95% CI, 1.15–1.95; *P* = .003).^[17]^

### 4.6. Certainty in the estimates

The included studies were observational except for the trial conducted by Pivot et al with variable methodological quality resulting in an increased risk of selection and confounding bias. The estimates were precise for baseline LVEF but not for other predictors, given a smaller number of events. There was no indirectness or evidence of publication bias. Studies were heterogeneous as they included patients with variable malignancies, co-morbidities, and baseline LVEF. Overall, the certainty in the estimates in all the 5 predictors was judged to be low.

## 5. Discussion

This systematic review identified LVEF as the most utilized echocardiographic measure in the literature to predict symptomatic cardiotoxicity. However, it may not be the most accurate. We demonstrate that baseline LVEF is inconsistent in predicting symptomatic cardiotoxicity in patients undergoing chemotherapy for malignancies. This may partly be due to the variable loading conditions and physiologic conditions such as heart rate, blood pressure, anemia, volume status, and fever that can all influence LVEF. Studies have identified that applying automated echocardiography techniques using deep learning algorithms and artificial intelligence can increase the accuracy of LVEF measurement and improve disease diagnosis. However, these techniques are not commonly applied in clinical settings.^[21,22]^ Myocardial deformation indices, particularly GLS, can detect changes in systolic dysfunction earlier in comparison with LVEF. Baseline GLS also appears to be a better predictor of symptomatic cardiotoxicity and overall mortality, as identified by this meta-analysis. Other echocardiographic parameters such as LVEDV, LVESV, and LVEDVI have been reported in studies for predicting symptomatic cardiotoxicity. The results from these measures of LV dimension have been mixed with no clear association with symptomatic cardiotoxicity.

We did not find a similar systematic review or meta-analysis performed previously. This is likely due to the scarcity of data regarding this topic. Oikonomou et al published a meta-analysis of the prognostic value of GLS for chemotherapy-related cardiotoxicity but included studies with patients having both symptomatic and asymptomatic cardiac dysfunction related to chemotherapy.^[13]^ Therefore, a relationship solely to symptomatic cardiotoxicity could not be ascertained. Like our study, Oikonomou et al identified that GLS had an excellent prognostic performance for subsequent chemotherapy-related cardiac dysfunction. Several other studies have established an association of lower absolute GLS values with a risk of developing chemotherapy-induced cardiotoxicity, both before and during treatment.^[23–25]^ Whether or not these results can be extrapolated to symptomatic cardiotoxicity remains unclear.

Most of the studies included in this analysis are nonRCTs or observational studies. Therefore, the scientific evidence is not of the highest quality. The question of whether echocardiography can predict clinical cardiotoxicity remains challenging. This is because of the wide variance in the results reported from observational and randomized trials conducted on this topic. Furthermore, the American Society of Echocardiography and the European Society of Cardiovascular Imaging Guidelines for measuring LVEF using the Simpson volumetric biplane method are not always employed in LVEF assessment.^[26]^ Adopting society guidelines and moving to a more accurate and reproducible 3D LVEF may overcome some of these challenges.^[27]^

Our analysis demonstrated that baseline GLS has a significant association with symptomatic cardiotoxicity. The importance of GLS in predicting cardiotoxicity has been under-investigated to date. Ali et al found that a prechemotherapy GLS value of less than an absolute value of –17.5% before anthracycline treatment would have correctly identified 24 out of the 28 patients who eventually developed symptomatic cardiotoxicity.^[9]^ Additionally, GLS can predict symptomatic cardiotoxicity in patients with normal LVEF. Similarly, Mousavi et al prospectively studied a patient population with normal EF prior to the initiation of anthracycline chemotherapy.^[19]^ GLS was found to be highly predictive of major adverse cardiac events (MACE), and when GLS was added to any other clinical variable, the prognostic value increased. Furthermore, GLS was predictive of symptomatic cardiotoxicity even in patients with a normal EF (50–59%), highlighting the role of earlier detection prior to a clinically detectable reduction in EF.^[8]^ GLS may be more accurate than LVEF since it is measured using automated software with less human interference. The values are reproducible if the same software is utilized for measurement.^[28,29]^

Our meta-analysis did not show a significant association between baseline LVEF and symptomatic cardiotoxicity. Nevertheless, LVEF is the most utilized echocardiographic measurement to monitor cardiac function in patients undergoing chemotherapy.^[2]^ Sawaya et al studied the role of LVEF and LV dimensions in predicting symptomatic cardiotoxicity in patients receiving chemotherapy with anthracyclines, taxanes, and trastuzumab.^[30]^ It was noted that LVEF measured at the completion of anthracycline treatment or changes in LVEF between baseline and completion of anthracycline treatment were not predictive of later cardiotoxicity. Separately, Mousavi et al prospectively studied a patient population with normal EF before the initiation of anthracycline chemotherapy.^[19]^ The authors found no statistically significant association between LVEF and the occurrence of MACE.^[19]^ These results were echoed in multiple other studies.^[10,15,16,19]^ Ali et al, however, noticed that individuals with decreased LVEF prior to chemotherapy had a greater risk of subsequent cardiac events.^[9]^ Similarly, Pivot et al noted that LVEF < 55% before initiation of trastuzumab corresponded to a higher risk of later cardiac events.^[18]^

## 6. Limitations

This meta-analysis has certain limitations primarily due to limitations in the included trials. Apart from 1 trial performed by Pivot et al, all the other studies were observational, introducing the possibility of selection and sample biases. The studies are heterogeneous, as they enrolled patients with different malignancies and were treated with different chemotherapeutic regimens. The method of measuring echocardiographic parameters was not similar in the included trials. The universal definition of cardiotoxicity seems to be missing, and the studies included in this analysis described cardiotoxicity differently. Many studies included an asymptomatic decline in LVEF and GLS as cardiotoxicity; therefore, they were excluded. This limited the number of studies included in this analysis, particularly for GLS, which affects the generalizability of the results. The baseline cardiac characteristics and primary endpoints also differed between studies, and the data often did not include the number of events. As a result, the meta-analysis was performed using hazard ratios and confidence limits.

## 7. Conclusions

Despite conflicting studies regarding the ability of LVEF reduction to predict symptomatic cardiotoxicity in patients receiving chemotherapy, LVEF assessment has remained the most used modality in predicting adverse clinical events in these patients. The data regarding GLS association with symptomatic cardiotoxicity in chemotherapy is encouraging. The studies examining this modality demonstrated that GLS was predictive of symptomatic adverse cardiac events, even in those patients with normal LVEF. Still, these results need further confirmation by RCTs studying the relationship between symptomatic cardiotoxicity and GLS. Further RCTs comparing GLS versus LVEF would be revealing. Global longitudinal strain is now automated and available on most commercial echocardiogram equipment and can also be performed offline using vendor-neutral software. Though normative values vary based on vendors, this should not be a significant issue if the individual patient is followed serially using the same product.

This study offers insight into the value of GLS in the risk stratification of patients receiving or who are about to receive chemotherapy, particularly anthracycline or trastuzumab-based regimens. Early detection of injury and subsequent intervention offers patients the best outcomes immediately and in the long term. In addition, patients deemed to be at higher risk of developing symptomatic cardiotoxicity can be monitored more closely, particularly with GLS pre-and postchemotherapy, with the addition of cardioprotective agents and cardiology consultation whenever clinically appropriate.

### Author contributions

MS design, data collection, manuscript, supervision. YY data collection, analysis, manuscript. HH data collection, analysis, manuscript. JJ data collection, analysis, manuscript. AP data collection, analysis, manuscript. MS data collection, analysis, manuscript, supervision.

## Supplementary Material


