# Supplementary material for: Echocardiographic predictors of symptomatic cardiotoxicity among patients undergoing chemotherapy: A systematic review and meta-analysis
Source: Medicine (Baltimore). 2022 Jul 15;101(28):e29562. doi: 10.1097/MD.0000000000029562 (PMC11132334; doi:10.1097/MD.0000000000029562)
Supplement: Supplementary file 1 [file medi-101-e29562-s001.pdf]

Supplementary Figure 1: Combination of keywords for search strategy

# ▲	Searches
1	Echocardiography/ or global longitudinal strain.mp.
2	Ventricular Function, Left/ or Echocardiography, Doppler/ or myocardial strain.mp.
3	echocardiographic characteristics.mp.
4	1 or 2 or 3
5	chemotherapy.mp. or Drug Therapy/
6	ANTHRACYCLINES/
7	5 or 6
8	Ventricular Dysfunction, Left/
9	Myocardial Ischemia/ or Myocardial Infarction/ or cardiac events.mp.
10	cardiac death.mp.
11	Heart Failure/
12	8 or 9 or 10 or 11
13	4 and 7 and 12
14	limit 13 to (english language and humans)
15	limit 14 to (clinical study or clinical trial, all or clinical trial, phase i or clinical trial, phase ii or clinical trial, phase iii or clinical trial, phase iv or clinical trial or controlled clinical trial or guideline or journal article or observational study or practice guideline or randomized controlled trial)

Supplementary Table 1: Risk of bias assessment

Author, Year	Selection Bias	Attrition Bias	Performance Bias	Detection Bias	Reporting Bias	Overall Bias
Ali, 2016	High	Low	High	Low	Low	Low
Mousavi, 2015	High	High	High	Low	Low	Low
Pivot, 2015	Low	Low	Low	Low	Low	Low
Wang, 2015	High	Low	High	Low	Low	Low

Supplementary Table 2: Echocardiographic predictors of symptomatic cardiotoxicity with hazard ratios and p-values.

Author, Year	Echo Parameters	N(n)	HR(CI)	P value
Ali, 2016		450(28)		
	LVEF		0.93(0.89-0.98)	.005
	LVEDV		0.99(0.98-1.01)	.580
	LVEDVI		1.00(0.97-1.03)	.910
	LVESV		1.01(0.98-1.04)	.370
	LVESVI		1.05(0.99-0.11)	.058
	GLS		1.47(1.35-1.59)	.000
	ASLS		1.85(1.65-2.08)	.000
Mousavi, 2015		158(12)		
	LVEF		0.88(0.71-1.06)	0.19
	LVEDV		1.03(1.01-1.05)	0.012
	LVEDVI		1.04(1.00-1.09)	0.039
	LVESV		1.07(1.02-1.12)	0.0029
	LVESVI		1.11(1.02-1.20)	0.012
	GLS		1.36(1.10-1.70)	0.0065
Pivot, 2015		3380(178)		
	LVEF<55	161(34)	5.12(3.51-7.45)	0.0001
	LVEF>55	3155(144)		
Wang, 2015		2285(65)		
	LVEF		1.38(1.19-1.59)	<0.0001
	LVID		1.38(.108-1.76)	<0.0001
	LVIS		1.50(1.15-1.95)	0.003

Legend: *LVEF* Left Ventricular Ejection Fraction, *LVEDV* Left Ventricular End Diastolic Volume, *LVESV* Left Ventricular End Systolic Volume, *LVEDVI* Left Ventricular End Diastolic Volume Index, *LVESVI* Left Ventricular End Systolic Volume Index, *GLS* Global Longitudinal Strain, *ASLS* Average Segmental Longitudinal Strain

Supplementary Table 3: SIGN risk of bias methodology for the included observational studies.

Questions	Ali, 2016	Mousavi, 2015	Wang, 2015
The study addresses an appropriate and clearly focused question.	Yes	Yes	Yes
The two groups being studied are selected from source populations that are comparable in all respects other than the factor under investigation.	NA	NA	NA
The study indicates how many of the people asked to take part did so, in each of the groups being studied.	NA	No	NA
The likelihood that some eligible subjects might have the outcome at the time of enrolment is assessed and taken into account in the analysis.	Yes	Yes	Yes
What percentage of individuals or clusters recruited into each arm of the study dropped out before the study was completed.	Yes	No	NA
Comparison is made between full participants and those lost to follow up, by exposure status.	NA	No	NA
The outcomes are clearly defined.	Yes	Yes	Yes
The assessment of outcome is made blind to exposure status. If the study is retrospective this may not be applicable.	NA	Yes	NA
Where blinding was not possible, there is some recognition that knowledge of exposure status could have influenced the assessment of outcome.	Yes	Yes	No
The method of assessment of exposure is reliable.	Yes	Yes	Yes

Evidence from other sources is used to demonstrate that the method of outcome assessment is valid and reliable.	Yes	Yes	Yes
Exposure level or prognostic factor is assessed more than once.	No	Yes	No
The main potential confounders are identified and taken into account in the design and analysis.	Yes	Yes	Yes
Have confidence intervals been provided?	Yes	Yes	Yes

Supplementary Table 4: Cochrane risk of bias tool for the included randomized controlled trials.

Author, Year	Sequence Generation	Allocation Concealment	Blinding Participants	Blinding Assessors	Incomplete Outcome Data	Selective Reporting	Other Bias
Pivot, 2015	NA	Yes	Yes	Yes	No	No	None
